# Inter-observer and intra-observer variability in immunohistochemical detection of endometrial stromal plasmacytes in chronic endometritis

**DOI:** 10.3892/etm.2012.824

**Published:** 2012-11-22

**Authors:** KOTARO KITAYA, TADAHIRO YASUO

**Affiliations:** 1 Department of Anatomy and Cell Science, Kansai Medical University, Osaka 570-8506;; 2 Department of Obstetrics and Gynecology, Graduate School of Medical Science, Kyoto Prefectural University of Medicine, Kyoto 602-8566, Japan

**Keywords:** CD138, chronic endometritis, plasmacyte, inter-observer agreement, intra-observer agreement

## Abstract

Chronic endometritis (CE) is an unusual endometrial inflammation characterized by stromal plasmacyte infiltration. CE is easily missed due to its subtle symptoms and demanding histopathological examinations. Although the immunohistochemistry for the plasmacyte marker CD138 has facilitated the detection of endometrial stromal plasmacytes, the accuracy and biases of this method for CE diagnosis remain poorly understood. The aim of this study was to investigate the inter- and intra-observer variability in the immunohistochemical detection of stromal plasmacytes in the human endometrium. A total of 80 CE and 20 non-pathological archival hematoxylin-stained endometrial preparations with or without immunostaining for CD138 were evaluated independently by two experienced observers and two inexperienced observers. Endometrial stromal plasmacytes in unit areas were counted in the hematoxylin-stained and CD138-immunostained preparations. Each preparation was subdivided into 11 categories by every five plasmacyte counts. The second evaluation was performed four weeks after the first evaluation. The immunohistochemical detection method was superior to conventional histopathological evaluation in both the inter- and intra-observer agreement, irrespective of the experience level of the observers. The linear weighted κ coefficient for intra-observer agreement was higher in the experienced observers than in the inexperienced observers. The inter-observer agreement among the four observers by the immunohistochemical detection method was similarly good between the first and second evaluation. There was no significant inter- or intra-observer variability in the paired comparison of the individual samples. These findings validate the use of immunohistochemistry for CD138 as an accurate and less biased diagnostic tool for CE.

## Introduction

Chronic endometritis (CE) is an inflammatory condition characterized by endometrial stromal plasmacyte infiltration which are unusual leukocyte populations in this mucosal tissue. CE is easily missed in gynecological practice, as it is typically asymptomatic or oligosymptomatic with mild manifestations of leukorrhea, pelvic discomfort or slight vaginal bleeding. Even symptomatic CE may be overlooked since endometrial sampling and histopathological detection of plasmacytes are required for diagnosis. In addition, endometrial stromal plasmacytes are difficult to detect using conventional tissue staining techniques (1).

Studies have demonstrated that CE is associated with female reproductive disorders, including repeated embryo implantation failure following *in vitro* fertilization-embryo transfer, unexplained infertility and unexplained recurrent miscarriages (2–5). The accurate diagnosis of CE is therefore a demanding task. The cell surface heparan sulfate proteoglycan CD138 (also known as syndecan-1) has been utilized in the diagnosis of plasmacytoma as a specific and sensitive marker of bone marrow and circulating blood malignant plasmacytes (6). Immunohistochemistry for CD138 has also been observed to be suitable for the detection of endometrial stromal plasmacytes in paraffin-embedded specimens and improves the diagnosis rate of CE (1,7,8).

The diagnostic accuracy and biases of CE in immunohistochemistry, however, remain poorly understood. Few reports have investigated the diagnostic variability for a single observer and between multiple observers. In the present study, we aimed to compare the inter- and intra-observer variability between the immunohistochemical and conventional histopathological methods in the detection of endometrial stromal plasmacytes.

## Materials and methods

### Specimens

A total of 100 archival preparations of endometrial specimens were used for the study under the approval of the local ethics committee. All these specimens were previously stained with hematoxylin with or without immunostaining using a primary anti-human CD138 monoclonal IgG antibody B-A38 (Nichirei Corp., Tokyo, Japan) and labeled streptavidin-biotin method (Dako, Kyoto, Japan). Of them, 80 specimens were diagnosed with CE by two experienced observers of human endometrial specimens, whereas the remaining 20 specimens were diagnosed as non-pathological (9).

### Immunohistochemical and histopathological analysis

Under a light microscope (magnification, x400), the preparations were evaluated independently by two experienced observers and two inexperienced undergraduate medical trainees unconnected with the study. Instruction was given to the inexperienced observers on the basic endometrial morphology, cell components and appearance of plasmacytes. In the hematoxylin-stained preparations, endometrial stromal plasmacytes were counted in 20 non-overlapping stromal areas according to the nuclear chromatin rearrangement which appeared as clock-face or spoke-wheel patterns. In the CD138-immunostained preparations, the number of immunoreactive cells was counted in the same number of stromal areas. The observers were blinded to each other’s findings until every observer had finished their evaluations. Each preparation was subdivided into 11 categories by every five cell counts. The second evaluation was performed 4 weeks after the first.

### Statistical analysis

The inter-observer or intra-observer variability was calculated using weighted and Fleiss’ κ statistics. The multiple comparisons between the datasets were performed with Tukey’s test. The intra-observer variability for an individual was compared using the Wilcoxon signed-rank test. P<0.05 was considered to indicate a statistically significant difference.

## Results

### Duration of the diagnostic evaluation

Under the conventional histopathological method, the inexperienced observers (3 and 4) failed to complete the full evaluation of all 100 preparations within one week. By contrast, they were able to identify and enumerate endometrial CD138^+^ stromal plasmacytes in all preparations using the immunohistochemical method. The duration required for microscopic evaluation per preparation in the immunohistochemical method was similar (P>0.66) between the experienced (mean ± SD, 2.4±1.1 and 2.6±0.8 min for observers 1 and 2, respectively) and inexperienced observers (2.8±1.2 and 2.3±1.3 min for observers 3 and 4, respectively).

### Inter-observer variability

In the 11-tier evaluation system, the linear weighted κ coefficient for inter-observer agreement was higher in the immunohistochemical method (0.968–0.976) than in the conventional histopathological method (0.687–0.771), irrespective of the experience level of the observers (Tables I and III). The inter-observer agreement among the four observers in the immunohistochemical method was similarly good in both the first and second evaluations (Fleiss’ κ coefficients, 0.833 and 0.861, respectively).

### Intra-observer variability

The immunohistochemical method showed good intra-observer agreement (linear weighted κ coefficient 0.933–0.968) for the experienced and inexperienced observers (Tables II and IV), in contrast to the conventional histopathological method, which showed fair agreement (0.477–0.658). The linear weighted κ coefficient for intra-observer agreement was higher in the experienced observers than in the inexperienced observers. There was no significant inter- (P= 0.57) or intra–observer variability (P=0.67) in the paired comparison of the individual samples.

## Discussion

In the present study, favorable inter- and intra-observer agreements for the detection of endometrial stromal plasmacytes using immunohistochemistry for CD138 were demonstrated. The inexperienced observers who failed to complete the full evaluation using the conventional histopathological method were able to detect and count plasmacytes easily by the immunohistochemical method, confirming the superiority of this approach for objectivity and reproducibility in the diagnosis of CE.

By contrast, the conventional histopathological method showed a certain degree of intra-observer variability. Bayer-Garner and Korourian reported that the diagnosis rate of CE by the conventional histopathological method (15%) was significantly lower than that by the immunohistochemical method (42%), in endometrial curettage samples (1). The low detection rate by the conventional histopathological method is likely to result from a high degree of interference in the distinction of local plasmacytes which morphologically resemble stromal fibroblasts or mononuclear cells (1,9).

One major concern in the sole immunohistochemical detection of CE is that the representative clones of anti-human CD138 antibodies detect not only stromal plasmacytes but also endometrial glandular/surface epithelial cells which constitutively express this proteoglycan. Although CD138 immunostaining in endometrial epithelial cells is weaker than in the stromal plasmacytes in the CE samples (10), the conditions of the preparations may potentially be misleading in certain CE specimens. For instance, an isolated or detached glandular epithelial cell may be misidentified as a stromal plasmacyte, leading to an overestimation of plasmacyte infiltration. The combined use of immunohistochemistry and conventional hematoxylin staining appears to reduce these types of errors.

In conclusion, immunohistochemistry for CD138 is a convenient and reliable tool for the detection of endometrial stromal plasmacytes in CE. Wider use of this methodology is expected to improve diagnostic accuracy, simplify testing and reduce examiner bias.

## Figures and Tables

**Table I.** Representative inter-observer agreement (between experienced observer 1 and inexperienced observer 3) on endometrial stromal CD138^+^ plasmacyte density by the immunohistochemical method.

Endometrial stromal CD138^+^ plasmacyte density: observer 1 (experienced):	Endometrial stromal CD138^+^ plasmacyte density: observer 3 (inexperienced)										
	0–4	5–9	10–14	15–19	20–24	25–29	30–34	35–39	40–44	45–49	≥50
0–4	16	3	0	0	0	0	0	0	0	0	0
5–9	2	**11**	**1**	**0**	**0**	**0**	**0**	**0**	**0**	**0**	**0**
10–14	0	**1**	**10**	**2**	**0**	**0**	**0**	**0**	**0**	**0**	**0**
15–19	0	**0**	**1**	**7**	**0**	**0**	**0**	**0**	**0**	**0**	**0**
20–24	0	**0**	**0**	**0**	**5**	**0**	**0**	**0**	**0**	**0**	**0**
25–29	0	**0**	**0**	**0**	**1**	**3**	**2**	**0**	**0**	**0**	**0**
30–34	0	**0**	**0**	**0**	**0**	**1**	**3**	**1**	**0**	**0**	**0**
35–39	0	**0**	**0**	**0**	**0**	**0**	**0**	**2**	**1**	**1**	**0**
40–44	0	**0**	**0**	**0**	**0**	**0**	**0**	**1**	**4**	**1**	**1**
45–49	0	**0**	**0**	**0**	**0**	**0**	**0**	**0**	**1**	**5**	**2**
≥50	0	**0**	**0**	**0**	**0**	**0**	**0**	**0**	**1**	**1**	**9**

Plasmacyte density was calculated as immunoreactive cell counts in 20 non-overlapping stromal areas and subdivided into 11 categories by every five cell counts. Bold numbers indicate the number of cases where the two independent observers agreed on the presence of CE. Underlined numbers indicate the number of cases where the two observers disagreed on the CE diagnosis. Numbers that are neither bold nor underlined indicate the numbers of cases where the two independent observers agreed on the absence of CE.

**Table II.** Representative intra-observer agreement (within the results for inexperienced observer 4) on endometrial stromal CD138^+^ plasmacyte density.

Endometrial stromal CD138^+^ plasmacyte density: observer 4 (first evaluation)	Endometrial stromal CD138^+^ plasmacyte density: observer 4 (second evaluation)										
	0–4	5–9	10–14	15–19	20–24	25–29	30–34	35–39	40–44	45–49	≥50
0–4	17	2	0	0	0	0	0	0	0	0	0
5–9	1	**11**	**2**	**0**	**0**	**0**	**0**	**0**	**0**	**0**	**0**
10–14	0	**1**	**11**	**1**	**0**	**0**	**0**	**0**	**0**	**0**	**0**
15–19	0	**0**	**0**	**7**	**1**	**0**	**0**	**0**	**0**	**0**	**0**
20–24	0	**0**	**0**	**0**	**5**	**0**	**0**	**0**	**0**	**0**	**0**
25–29	0	**0**	**0**	**0**	**1**	**4**	**1**	**0**	**0**	**0**	**0**
30–34	0	**0**	**0**	**0**	**0**	**1**	**4**	**0**	**0**	**0**	**0**
35–39	0	**0**	**0**	**0**	**0**	**0**	**0**	**3**	**1**	**0**	**0**
40–44	0	**0**	**0**	**0**	**0**	**0**	**0**	**0**	**6**	**1**	**0**
45–49	0	**0**	**0**	**0**	**0**	**0**	**0**	**0**	**1**	**6**	**1**
≥50	0	**0**	**0**	**0**	**0**	**0**	**0**	**0**	**0**	**1**	**10**

Bold numbers indicate the number of cases where the observer diagnosed the presence of CE in the first and second evaluation. Underlined numbers indicate the number of cases where the CE diagnosis at the first and second evaluations did not agree. Numbers that are neither bold nor underlined indicate the number of cases where the observer diagnosed the absence of CE in the first and second evaluations.

**Table III.** Linear weighted κ values for inter-observer variability of the first endometrial stromal CD138^+^ plasmacyte density evaluation using the immunohistochemical method and conventional histopathological method (in parenthesis).

Observer	Observer 1	Observer 2	Observer 3
Observer 2	0.976 (0.771)	-	-
Observer 3	0.973 (0.701)	0.968 (0.719)	-
Observer 4	0.973 (0.725)	0.968 (0.729)	0.973 (0.641)

**Table IV.** Linear weighted κ values for intra-observer variability of endometrial stromal CD138^+^ plasmacyte density evaluation using the immunohistochemical method and conventional histopathological method (in parenthesis).

	Second evaluation			
First evaluation	Observer 1	Observer 2	Observer 3	Observer 4
Observer 1	0.965 (0.649)	-	-	-
Observer 2	-	0.968 (0.658)	-	-
Observer 3	-	-	0.933 (0.477)	-
Observer 4	-	-	-	0.939 (0.515)
